# Supplementary material for: Gαq-Stimulated Gene Expression Is Insensitive to Bromo Extra Terminal Domain Inhibitors in HEK 293 Cells
Source: Int J Mol Sci. 2025 Sep 12;26(18):8904. doi: 10.3390/ijms26188904 (PMC12470112; doi:10.3390/ijms26188904)
Supplement: Supplementary file 1 [file ijms-26-08904-s001.zip › ijms-3825196-supplementary.pdf]

Supplementary Materials

Gαq-stimulated gene expression is insensitive to Bromo extra terminal domain inhibitors in HEK 293 cells

Ashika Jain, Viviane Pagé, Dominic Devost, Darlaine Pétrin, Terence E. Hébert*, Jason C. Tanny*

Department of Pharmacology and Therapeutics, McGill University, Montréal, Québec, H3G 1Y6, Canada
* Correspondence: jason.tanny@mcgill.ca (JCT); terence.hebert@mcgill.ca (TEH)

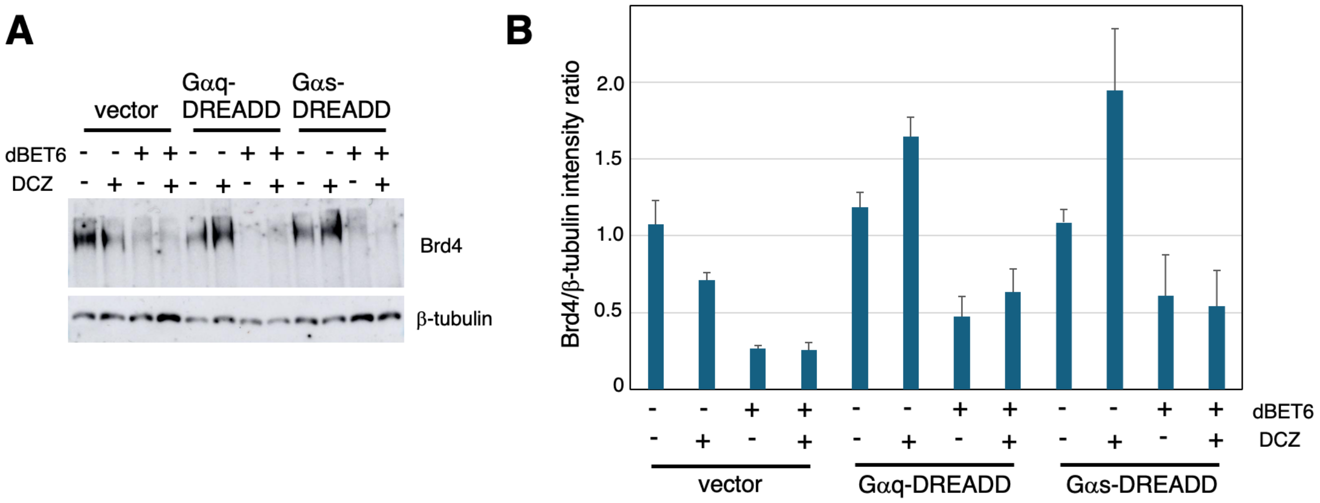

Figure S1. *Brd4 depletion by dBET6 in presence and absence of DREADD signaling.* **(A)** Representative immunoblot of extracts from HEK 293 cells transfected with the indicated plasmids and treated with dBET6 (100 nM for 3 hours), DCZ (1 μM for 1 hour), or both (with DCZ treatment occurring in the last hour of dBET6 treatment). Antibodies are indicated on the right. **(B)** Quantification of Brd4 signals normalized to β-tubulin under the indicated conditions (n=3; error bars indicate SEM).

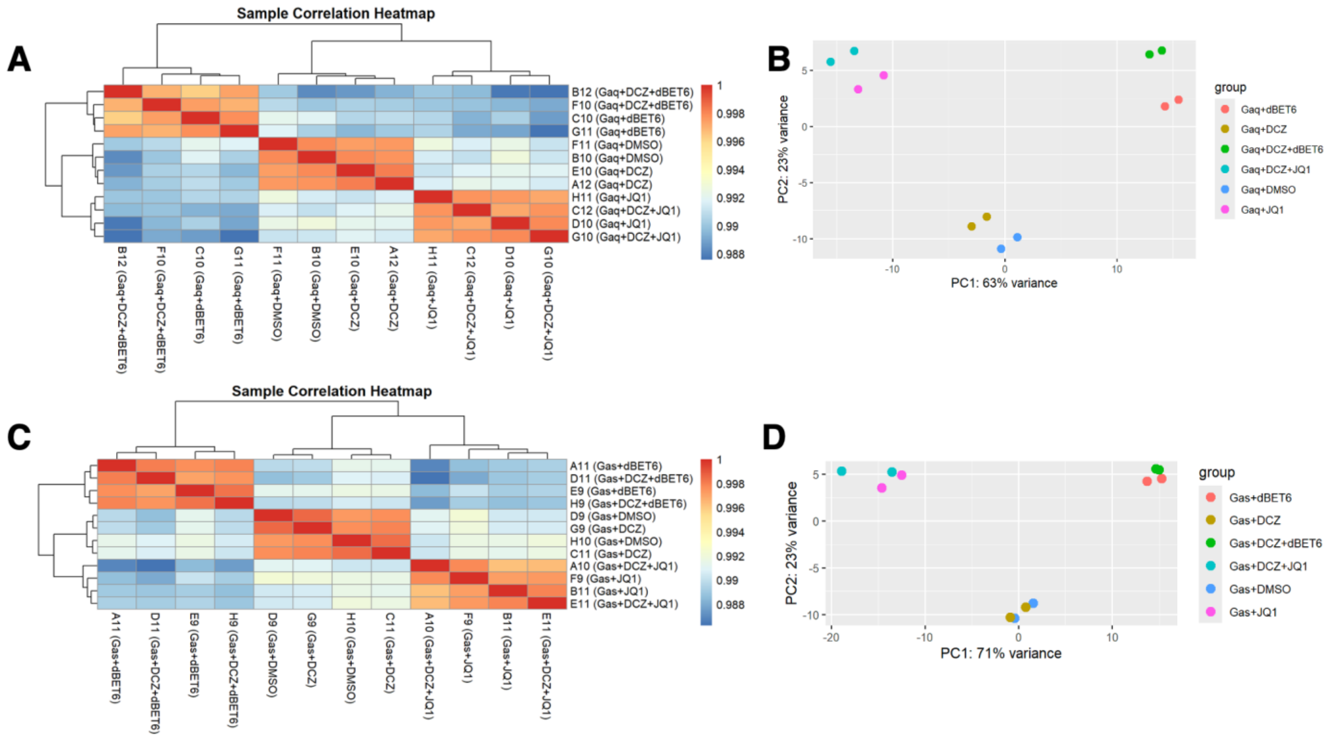

Figure S2. *Pearson correlations and principal component analysis for RNA-seq datasets*. **(A)** Pearson correlation heatmap for RNA-seq datasets from Gαq-DREADD expressing cells. P-values indicated by the colour bar. **(B)** Principal component analysis of RNA-seq datasets from Gαq-DREADD expressing cells. **(C)** Pearson correlation heatmap for RNA-seq datasets from Gαs-DREADD expressing cells. P-values indicated by the colour bar. **(D)** Principal component analysis of RNA-seq datasets from Gαs-DREADD expressing cells.

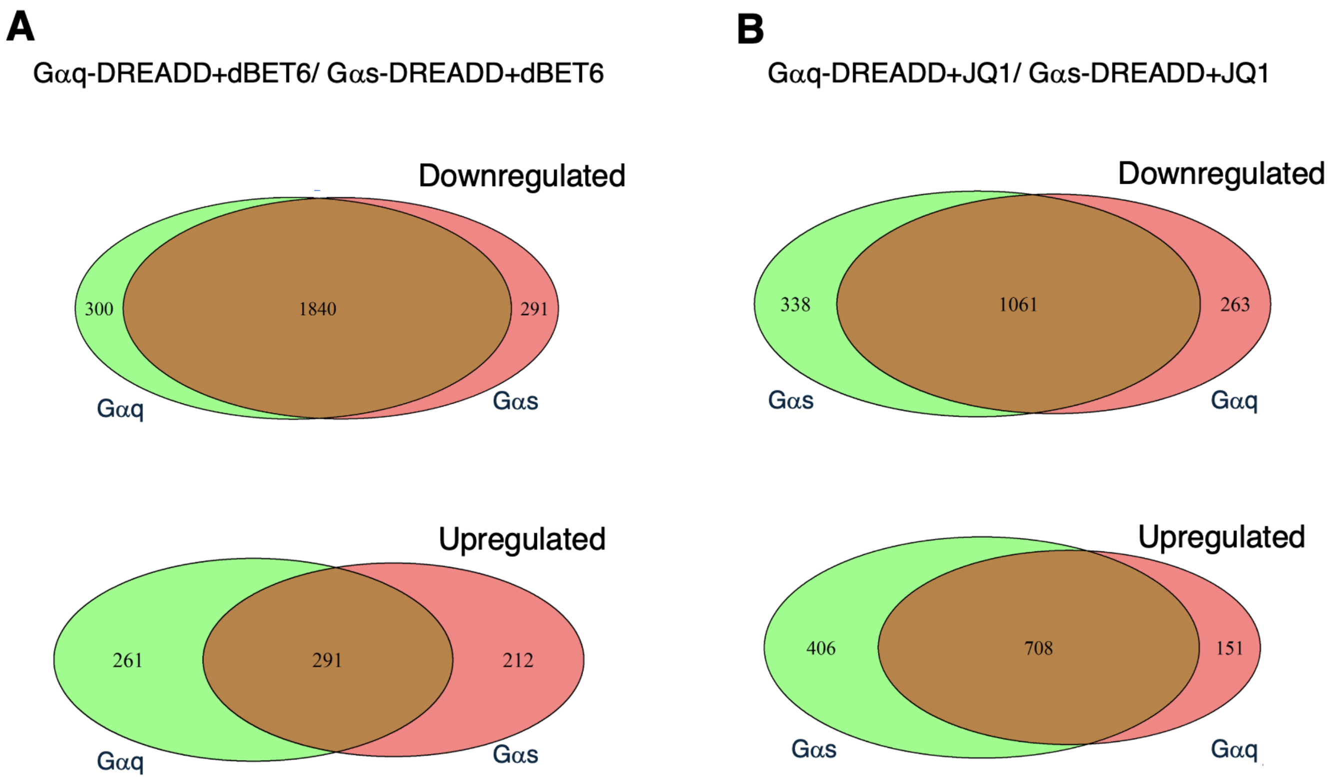

Figure S3. *Comparison of the effect of BET inhibitors in cells expressing Gαq-DREADD and Gαs-DREADD in the absence of DCZ.* **(A)** Venn diagrams comparing the significantly downregulated (top) or upregulated (bottom) genes identified upon dBET6 treatment of cells expressing Gαq-DREADD or Gαs-DREADD. **(B)** Venn diagrams comparing the significantly downregulated (top) or upregulated (bottom) genes identified upon JQ1 treatment of cells expressing Gαq-DREADD or Gαs-DREADD.

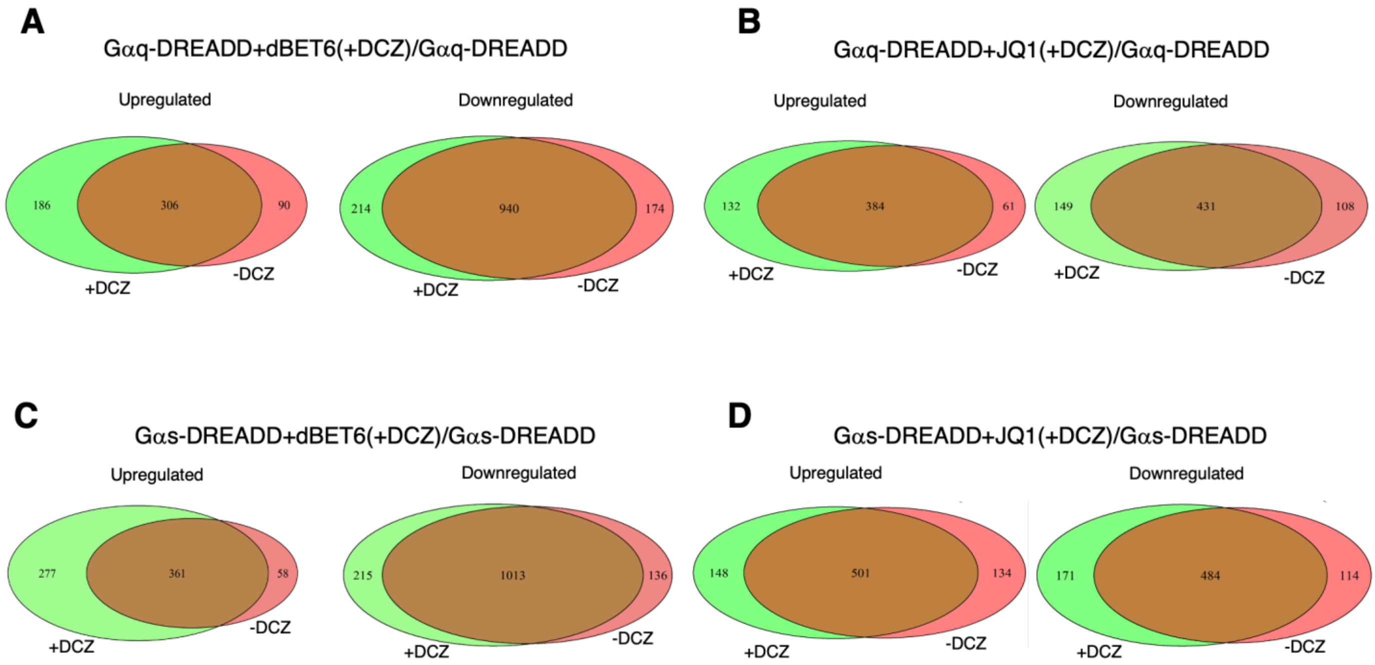

Figure S4. *Combined effects of DCZ and BET inhibitors*. **(A)** Venn diagrams comparing the significantly upregulated (left) or downregulated (right) genes identified upon combined DCZ/dBET6 treatment of cells expressing Gαq-DREADD. **(B)** Venn diagrams comparing the significantly upregulated (left) or downregulated (right) genes identified upon combined DCZ/JQ1 treatment of cells expressing Gαq-DREADD. **(C)** Venn diagrams comparing the significantly upregulated (left) or downregulated (right) genes identified upon combined DCZ/dBET6 treatment of cells expressing Gαs-DREADD. **(D)** Venn diagrams comparing the significantly upregulated (left) or downregulated (right) genes identified upon combined DCZ/JQ1 treatment of cells expressing Gαs-DREADD.

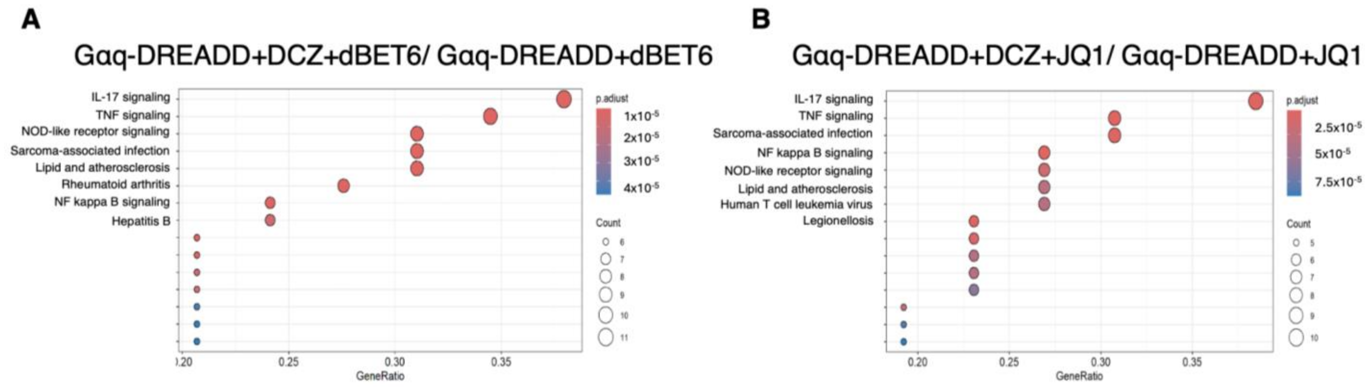

Figure S5. *Enrichment of inflammatory functions among genes upregulated by DCZ in Gαq-DREADD expressing cells in the presence of BET inhibitors*. **(A)** KEGG pathway analysis of 38 significantly upregulated genes in the presence of dBET6. **(B)** KEGG pathway analysis of 36 significantly upregulated genes in the presence of JQ1.

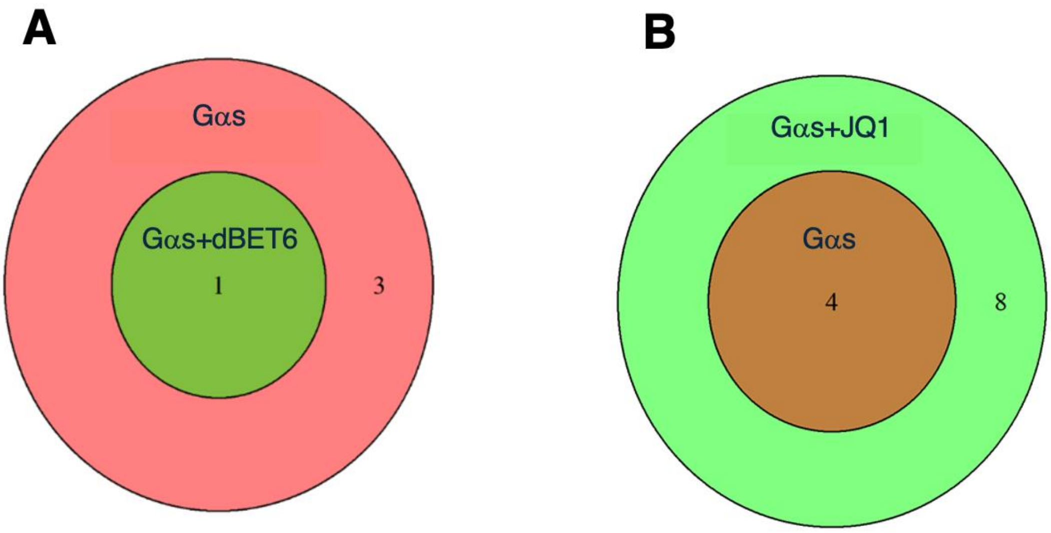

Figure S6. *Effects of BET inhibitors on Gαs-induced gene expression*. **(A)** Venn diagram comparing significantly upregulated genes identified upon DCZ treatment of cells expressing Gαs-DREADD in the presence or absence of dBET6. **(B)** Venn diagram comparing the significantly upregulated genes identified upon DCZ treatment of cells expressing Gαs-DREADD in the presence or absence of JQ1.
